# Automatic Detection of Pornographic and Gambling Websites Based on Visual and Textual Content Using a Decision Mechanism

**DOI:** 10.3390/s20143989

**Published:** 2020-07-17

**Authors:** Yang Chen, Rongfeng Zheng, Anmin Zhou, Shan Liao, Liang Liu

**Affiliations:** 1 College of Cybersecurity, Sichuan University, Chengdu 610065, China; chenyang652@163.com (Y.C.); zhouanmin@scu.edu.cn (A.Z.); liaoe1110@163.com (S.L.); 2 College of Electronics and Information Engineering, Sichuan University, Chengdu 610065, China; scu.ele.zrfeng@gmail.com

**Keywords:** website detection, decision mechanism, BoVW, Doc2vec, information processing, machine learning

## Abstract

Pornographic and gambling websites become increasingly stubborn via disguising, misleading, blocking, and bypassing, which hinder the construction of a safe and healthy network environment. However, most traditional approaches conduct the detection process through a single aspect of these sites, which would fail to handle the more intricate and challenging situations. To alleviate this problem, this study proposed an automatic detection system for porn and gambling websites based on visual and textual content using a decision mechanism (PG-VTDM). This system can be applied to the intelligent wireless router at home or school to realize the identification, blocking, and warning of ill-suited websites. First, Doc2Vec was employed to learn the textual features that can be used to represent the textual content in the hypertext markup language (HTML) source code of the websites. In addition, the traditional bag-of-visual-words (BoVW) was improved by introducing local spatial relationships of feature points for better representing the visual features of the website screenshot. Then, based on these two types of features, a text classifier and an image classifier were both trained. In the decision mechanism, a data fusion algorithm based on logistic regression (LR) was designed to obtain the final prediction result by measuring the contribution of the two classification results to the final category prediction. The efficiency of this proposed approach was substantiated via comparison experiments using gambling and porn website datasets crawled from the Internet. The proposed approach outperformed the approach based on a single feature and some state-of-the-art approaches, with accuracy, precision, and F-measure all over 99%.

## 1. Introduction

Nowadays, the rapid development of the Internet has paved the way for growing numbers of netizens to obtain and exchange information through websites. However, the information on the websites contains not only useful knowledge that people need but also a rapidly increasing amount of objectionable or illegal content, such as pornography, gambling, violence, and phishing. Due to a lack of supervision, a growing number of children and youngsters are exposed to these ill-suited websites. For instance, from 2018 to 2019, pornographic websites accounted for 4.28% of all websites accessed by Latin American children on personal computers (PCs), with a 3.65% increase compared to the previous year [1]. Pornographic and gambling websites cause great undesirable effects on the physical and psychological health of children [2]. They also greatly affect social harmony and increase the number of criminal cases to a certain extent [3]. Therefore, it is urgent and necessary for families and society to take effective measures to prevent teenagers from accessing these ill-suited websites.

To reduce the negative impact of these constantly appearing illegal websites, a variety of methods have been proposed to detect these websites. Early researchers often used blacklist-based approaches to detect illegal websites [4,5], which matched a suspicious domain with some pre-defined illegal domain in the blacklist. Despite its high processing speed, the negative aspect of this solution is that it cannot identify the illegal uniform resource locators (URLs) that are not in the blacklist in advance. Furthermore, due to the fast and irregular URL changes of illegal websites, it becomes a difficult task to update the newly launched illegal websites in the blacklist promptly [6].

Due to its active learning ability, machine learning has been applied to website categorization and has become a hotspot of illegal website detection. There are many practical solutions based on machine learning, which generally extract features from the URL [7] or the content of websites [8]. The URL-based illegal detection method only extracts features from the URL of the website with a short detection time. However, the URL cannot fully represent the features of the illegal website, and thus, sometimes URL-based approaches do not work well. Textual content is the main component of webpages, which is relatively rich and easy to obtain. Nevertheless, due to the increasing complexity and vastness of websites, text-based detection is vulnerable to the “curse of dimension” [9]. In recent years, many researchers have focused on using the visual content of web pages to classify websites and have made some achievements [10,11]. Unfortunately, the classification based on visual features is easily affected by the quality of the training set samples and the generalizability of models, resulting in a low recognition rate. For instance, some normal websites (underwear sales, medical websites, etc.) often display visual content similar to pornographic websites, leading to them being mistaken for pornographic websites [12]. Particularly, when the illegal websites conceal themselves via disguising, misleading, blocking, and bypassing, the traditional single-feature-detection methods may not be sufficient for website detection. Hybrid feature-based methods [13,14] are a more effective solution, which perform website detection by comprehensively analyzing multiple features of the website. However, if the combination of features is not appropriate, it will not only cause time consumption but also fail to obtain a satisfactory detection effect. As can be seen from the above analysis, the existing measures are weak in their ability to detect gambling and pornographic websites, and it is imperative to present an effective and accurate automatic detection approach to manage these intricate and changing problems.

To remedy the shortcomings of current website detection methods, we propose an effective system for the automatic detection of porn and gambling websites based on visual and textual content using a decision mechanism (PG-VTDM). In the proposed PG-VTDM, we extract two sorts of features from the hypertext markup language (HTML) source code and the website screenshot, including textual features learned using Doc2Vec and visual features learned using spatial bag-of-visual-words (Spa-BoVW). In particular, Spa-BoVW was obtained by introducing the local spatial relationship of website screenshots into the bag-of-visual-words (BoVW), which is more conducive to learning comprehensive and effective visual features. Then, the text-based classification and image-based classification were performed on these features, respectively. In the decision mechanism, the data fusion algorithm based on logistic regression (LR) was employed to fuse the classification results of the two classification models to give the final prediction category. By combining the information from the images and texts of the website and adopting a reasonable category decision mechanism, the ability of the proposed system could be maximized to detect gambling and pornography websites. The black and white list in the system was constantly updated in the detection process, greatly improving the detection speed.

The main contributions of this paper are listed as follows:

- An automatic detection system for porn and gambling websites was proposed in this study. Compared with the traditional single-feature-detection methods, this system achieved more accurate detection efficiency through comprehensively analyzing the textual features extracted from textual content in the HTML source code and the visual features extracted from the website screenshots. By maintaining and updating the black and white list libraries, the proposed system could greatly improve the detection speed while ensuring the detection performance.
- By introducing the spatial relation of feature points, we improved the traditional BoVW algorithm and propose the Spa-BoVW, which divides the website screenshots and fully considers the spatial position relationship of feature points between different blocks to achieve a more effective visual feature vector representation.
- An effective category decision mechanism was proposed for the final category prediction of the website. We applied a fusion algorithm based on LR to fuse the classification results of the text classifier and the image classifier. By adjusting the threshold value in the judgment condition, the detection performance of the method could be improved.
- The proposed method was comprehensively evaluated by conducting a large number of experiments on the porn and gambling dataset we collected. The experimental results show that our proposed method exhibited good competitive performance in terms of accuracy, precision, recall, and F-measure.

The remainder of this paper is organized as follows: Section 2 discusses related work. Section 3 introduces the framework and specific implementation of the system proposed in this paper. In Section 4, we verify the effectiveness of the proposed method via experiments and analyze the experimental results. Finally, Section 5 summarizes the paper and outlines future work.

## 2. Related Work

Booming illegal websites (such as gambling, pornography, and phishing websites) have drawn growing attention, and as such, multiple approaches have been developed to deal with illegal websites. These approaches can essentially be divided into three categories: content-based methods, URL-based methods, and hybrid feature-based methods. Subsequently, some representative achievements are summarized in the following section.

### 2.1. Content-Based Detection

Website content contains a wealth of information, such as text, images, links, cascading style sheets (CSS), and HTML tags. The content-based illegal detection methods take advantage of this information to detect illegal websites.

Early text-based methods treated the detection of illegal websites as a simple document classification task, only extracting features from the textual content (removing HTML tags, JavaScript, etc.) of the website [15,16]. For instance, Lee et al. [17] proposed an intelligent bilingual web page categorization engine to judge whether a web page contained pornographic content. The system employs a frequency count of keywords appearing in the text, along with the web page features to train neural networks. Although the system can achieve high accuracy, it is not suitable for real-time applications due to the high number of computations. In Ali et al. [12], a fuzzy-ontology/support vector machine (SVM)-based semantic knowledge system was proposed to identify and block access to pornography. This system uses unsupervised linear techniques to extract meaningful keywords from the context of the web content, where ontology-based semantic knowledge and fuzzy logic are used to judge the type of web page being evaluated. Despite some success in text-based detection methods, there are a few problems with these methods, as discussed by Hu et al. [18]. For example, some websites deliberately misspell keywords to avoid having illegal content being detected (misspelled problem). In addition, the over-blocking problem is becoming more prominent. Therefore, textual features are often analyzed in combination with other features to improve detection performance.

Later on, some approaches to detect illegal websites using more textual information, such as hyperlinks [19,20] and HTML tags [21], were developed. For example, Jain et al. [20] proposed a phishing attack detection method based on hyperlinks. This method analyzes the hyperlinks in the HTML source code of the website and extracts 12 types of features from it, which are used for training machine learning models. The main advantages of this approach are that it is third-party independent and language independent. Another research direction in this field detects websites from the perspective of page structure (layout) and page overall appearance style. Most of the research in this direction relies on feature sets, such as the document object model (DOM) [22,23] and CSS [24], for illegal website detection. For example, Xu et al. [23] analyzed the structures of HTML source codes of websites and proposed a fraudulent website detection approach based on a tree kernel and an SVM with high precision.

In recent years, some works have shown the potential of analyzing the visual information of websites to detect illegal websites [9,10,25]. As an important part of the web page, visual content has become an analysis focus of content-based detection. The visual-based detection method extracts significant visual features from the visual content for classification by utilizing effective image processing techniques. For instance, the approach in Li et al. [9] used the bag-of-words (BoW) model to extract visual features from website screenshots, which were further used to identify gambling and porn websites. However, this method ignores the spatial relationship of feature points in website screenshots, which blurs the spatial difference of website screenshots to some extent. In another study, Phoka et al. [25] utilized the visual information of a login page for phishing website detection. In this method, a sub-image random placement method was designed for data augmentation, providing a great deal of data for the training of a convolutional neural network (CNN) model. This method can effectively detect a phishing attack that fakes the login page by extending, moving, or deleting certain components of the webpage, but it does not solve the problem of highly dynamic page detection. In particular, some methods are designed for detecting pornographic images with a skin detection technique [26,27]. For example, the system in Mahmoud et al. [28] identifies adult websites by detecting pornographic content in images and introduces skin and face detection technology to achieve the purpose of performance improvement.

### 2.2. URL-Based Detection

In addition to using the information present in the websites, some methods are designed to extract features from the information provided by the URL of the website, such as URL strings [29,30] and URL statistics rules [7,31] for website classification. This kind of method has relatively few applications and is mainly used to detect phishing websites. For example, Verma et al. [32] focused on proposing a fast and accurate URL-based phishing attack detection system. Their solution extracts character-based N-grams from URLs with high precision by using a confidence-weighted vector and an adaptive regularization weight. The model proposed in [33] detects phishing sites based on URL features, which has high accuracy and strong adaptability. This model uses the grey wolf optimizer (GWO) algorithm for remedying the weaknesses of the traditional backpropagation neural network (BPNN) and the accuracy of phishing website recognition is improved by the dual feature evaluation mechanism.

The URL-based illegal detection methods do not need to access the page content of a website before making a decision, and as such, it has the advantage of a fast detection speed. However, the limitation of this type of method is that the URLs contain relatively little information and cannot fully represent the characteristics of illegal websites.

### 2.3. Hybrid Features-Based Detection

With rapid iterations of a website, the content of the website is increasingly rich, which makes extracting features from a single perspective no longer sufficient to determine whether a webpage is illegal in today’s scenario. For this reason, many researchers are working on combining multiple features to detect websites [34].

Ahmadi et al. [35] proposed a pornographic web page detection system based on hierarchical structure classifiers, which achieves a classification rate of 95% by comprehensively analyzing the characteristics of visual, textual, and profile features. A two-stage extreme learning machine (ELM) framework based on the combination of rule, textual, Web, and URL features was presented for phishing websites detection [36]. At first, ELM was designed as a classifier to classify the textual content of websites. The linear combination model-based ensemble ELMs (LC-ELMs) was employed for constructing the hybrid feature-based classification model in the next stage. Jain et al. [37] detected phishing attacks by analyzing keywords, hyperlinks, and CSS layouts in web pages. The approach can accurately detect the source of the webpage and the phishing webpages. Li et al. [38] proposed a phishing webpage detection system based on two sets of features. This method extracts features from the URL and HTML source codes of webpages and designs a stacking model using the combination of the gradient boosting decision tree (GBDT), extreme gradient boosting (XGBoost), and light gradient boosting machine (LightGBM), which achieves the complementarity of different models and improves the performance of the detection of phishing web pages. Hybrid feature-based methods can achieve satisfactory effects because they analyze the website from multiple perspectives but the design of multiple features may cause a lot of time consumption. Furthermore, if the feature combination is not reasonable, it will also affect the final detection performance.

In conclusion, the many existing methods used to detect illegal websites listed above only use a single type of feature, such as URL, textual, or visual features. Compared with these single-feature detection methods, hybrid feature-based methods have better detection performance and broader development prospects. To this end, this study combined the textual and visual features of the website to detect gambling and pornographic websites. In addition, to improve the detection performance of the websites, the decision mechanism was used to predict the final category of the website by measuring the impact of the classification of different features.

## 3. System Design

This section discusses our proposed system in detail. We first outline the overall framework of the proposed website detection system and then describe more technical details about each module in the framework.

### 3.1. Overview of the Proposed System

**Figure 1:**
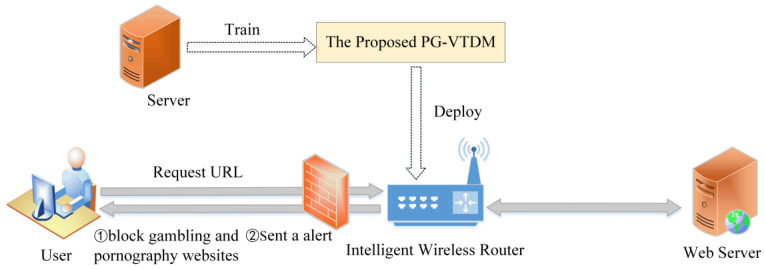
The working scene of the proposed system. PG-VTDM: Automatic detection system for porn and gambling websites based on visual and textual content using a decision mechanism, URL: Uniform resource locator.

Our system (PG-VTDM) was designed to automatically detect and filter gambling and pornographic websites on the Internet using an intelligent wireless router as the medium. Figure 1 shows the working scene of the proposed system. Usually, the user enters a URL in the browser to retrieve specific information. The intelligent wireless router deployed with the proposed PG-VTDM acts as a real-time illegal website monitor to check out the user’s access request and perform blocking. After receiving a user’s requested URL, the intelligent wireless router will systematically conduct detection for gambling and pornographic websites. If the web page is detected as being a gambling or pornography page, the smart wireless router will automatically block access to this website and sent an alert to the user in real time.

**Figure 2:**
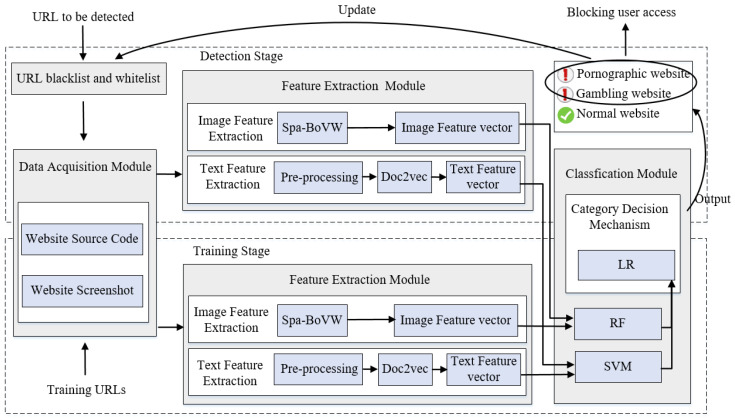
Overall framework of the proposed system. LR: Logistic regression, RF: Random forest, Spa-BOVW: Spatial bag-of-visual-words, SVM: Support vector machine.

Figure 2 presents the internal process and framework of the proposed system. Given a URL to be detected, the proposed system first compares this URL with the URL blacklist and whitelist libraries. If the URL already exists on the URL blacklist or whitelist library, the system identifies the website category directly without further content analysis. This step can greatly save the detection time of the system. After the blacklist and whitelist screening, for the detection of unknown URLs, the proposed web content filtering system will perform the following steps: (1) access the URL and then obtain the website source code and website screenshot of the corresponding website via web crawling and scrapping; (2) extract the textual and image features from website source code and website screenshots, respectively; (3) use the corresponding classifier to make preliminary predictions for these two types of features; (4) employ the decision mechanism to judge and output the final website category and then add this URL to our URL blacklist or whitelist for updating. For simplicity, the proposed framework is divided into three main processing modules, as follows:

#### 3.1.1. Data Acquisition Module

This module first analyzes the network data traffic requested by the user that is collected at the mirror of the egress router and obtains the URL of the website. Using the URL of the website, the website source code and website screenshots of the corresponding website are crawled by an automatic web crawling procedure.

#### 3.1.2. Feature Extraction Module

This module is divided into two sub-modules: image feature extraction and textual feature extraction. In the textual feature extraction module, the obtained website source code is preprocessed to remove the interference information in the text, and then the trained Dov2Vec is used to extract the textual features from the website source code. In the image feature extraction module, the traditional BoVW is improved by introducing the spatial relation of visual feature points, that is, the proposed Spa-BoVW is used to extract the image features of website screenshots.

#### 3.1.3. Classification Prediction Module

This module combines multiple machine learning models for classifying the given website. Based on the textual and visual features obtained in the feature extraction module, an SVM and a random forest (RF) are used as the text classifier and image classifier to classify the given website, respectively. Then, the category decision mechanism fuses the classification results of the two classifiers (SVM, RF) by using a fusion algorithm based on LR and produces a final prediction concerning the category of the website. Finally, based on the predicted website category, the system’s URL blacklist and/or whitelist is updated.

### 3.2. Website Content Acquisition

This phase aims to extract the raw information from a given website that needs to be analyzed, including the HTML source code and website screenshot.

First, the network data traffic between the user and the Web server is collected from the mirror of the egress router. Then, the protocol analysis tool is used for analyzing the data packet and obtaining the URL of the website. Based on the URL of the website, the web crawler was designed to capture the HTML source code and screenshots of the corresponding website. Although its operation seems simple, some problems can occur during web crawling and scrapping. For example, when using a browser to access a webpage, the crawler usually obtains only the source code of the website and the information loaded by JavaScript cannot be obtained. In addition, some illegal websites take a page jump to avoid detection, which means that the URL of the website eventually visited by users is not the same as the URL initially clicked. When taking a screenshot of a web page, it may result in the crawler only capturing the screenshot of the web page corresponding to the initial URL, but the screenshot of the target web site cannot be obtained correctly.

To solve the above problems, in this study, the WebDriver in the selenium library was used to launch the Chrome browser to achieve the effect of the dynamic rendering of website pages such that the crawler can obtain the complete website source code after loading the JavaScript code. Then, based on the corresponding interface function provided by WebDriver, the crawler can achieve the automatic scrolling of the slide-down box during the set time range and obtain the complete long screenshot corresponding to the final target website. It must be emphasized that, different from some image-based methods, we analyzed the visual information of the website from the perspective of website screenshots instead of extracting all the pictures on the website, mainly for the following three reasons. The first one is that the website screenshot contains more comprehensive visual content of the website, including the module layout, overall color style, etc., which may contribute toward extracting more effective visual features.

The second one is that there is a huge amount of work involved in characterizing all the images on the website, which will increase the burden of the detection system and some of which may produce interference information. The last one is that, when most of the images on the website do not reflect the actual type of websites (such as underwear sales websites and medical websites), analyzing website screenshots can help improve the detection performance.

### 3.3. Feature Extraction

#### 3.3.1. Textual Feature Extraction Using Doc2Vec

The pre-processing of the textual content on the website is a vital step, where its primary purpose is to remove unwanted noise and obtain the data in a specified format [39]. Because the website source code obtained via automatic web crawling not only contains the textual content relevant to the categorization but also inevitably contains a large amount of noise information, such as HTML/XML tags, hyperlinks, and JavaScript code, this noise content may negatively affect the performance in the classification phase and should be decreased as much as possible before the feature extraction phase. The pre-processing operations in this study were as follows:

- Removal of tags: There are many HTML tags (e.g., <div>…</div>, <span>…</ span >, <p>…</p>, etc.) that fill the HTML source code of the website. In this study, we combined regular expressions with Beautiful Soup to remove all the HTML tags from the HTML source code of the website. Moreover, the CSS and JavaScript in the HTML source code also needed to be removed.
- Removal of punctuation marks and special symbols: Punctuation marks and special symbols (e.g., =, %, &, etc.) are common but insignificant information in the text. The punctuation marks and other special symbols were replaced with space characters.
- Text segmentation: NLPIR-ICTCLAS2016 [40] was used for the Chinese word segmentation of the stripped textual content.
- Removal of stop words: Stop words are words that occur repeatedly in the text and their existence has no practical relevance to the categorization task. Furthermore, a large number of stop words will affect the performance of text classification. Common stop words include articles, prepositions, conjunctions, etc.

If the preprocessed data is directly transformed into feature vectors, it is likely to cause the dimension problem. Therefore, it is necessary to use a feature selection method to reduce the dimension of the feature space and improve the classification efficiency. Word2Vec [41] is a popular feature extraction method based on word embedding, which is effective at word-level presentation. It is important to note that Word2Vec is less adept at representing document-level text since it does not take into account the semantic relationship of the context. In this study, Doc2Vec [42] was used for learning a feature representation for a given document.

Doc2Vec is an unsupervised learning algorithm proposed by Quoc Le and Tomas Mikolov [42] and is based on Word2Vec, which shows perfect performance in the field of text mining [43,44,45]. There are two key advantages of using Do2vec: one is that it reserves the order and semantic information of the text when learning the vector representation of the text, while the other is that it learns the training data in an unsupervised environment such that it reduces the manual intervention to some extent. The essential idea of Doc2Vec is to learn fixed-length feature representations from variable-length text fragments (such as sentences, paragraphs, and documents) based on a neural network, that is, Doc2Vec can map text or sentences to dense real-valued vectors. In this way, the preprocessed document text of the website can be mapped into a k-dimensional space vector. Therefore, the semantic similarity of the textual content of the website can be represented by the similarity in vector space. The Doc2Vec algorithm has two different frameworks, namely, distributed memory model of paragraph vector (PV-DM) and distributed bag of words paragraph vector (PV-DBOW), the details of which are given below.

In the PV-DM framework, each document can be represented by a unique paragraph vector D in the vector space, while each word corresponds to the word vector W. Given the context word vectors and the paragraph vector, the PV-DM attempts to predict the next word in the context. Paragraph vectors can be used as memory stores to remember the topic of a paragraph or missing content from the current context. However, in the PV-DBOW framework, the document vector is considered as the only input feature, regardless of the input context word, and the model predicts words at random positions in the paragraph in the output. In this case, according to the given paragraph vector, the model samples a text window from the text at each iteration and then randomly samples a word from the window as the prediction task.

#### 3.3.2. Image Representation with Spa-BoVW

The bag-of-words (BoW) method was originally proposed in the field of text mining for information retrieval. Due to its simplicity and excellent performance, the BoW method became more popular and further expanded into the field of computer vision in the form of BoVW, which was applied to image classification [46], image retrieval [47], object recognition [48,49], etc. With the core idea similar to the BOW model, the BoVW model regards an image as a document, and the key features in the image are regarded as “words.” Although the traditional BoVW model is effective and easily applied to a wide range of image processing tasks, it has a well-known shortcoming: it overlooks location information among visual words in the image space. This may result in the loss of some useful spatial structure information of images, which affects the classification performance to some extent.

**Figure 3:**
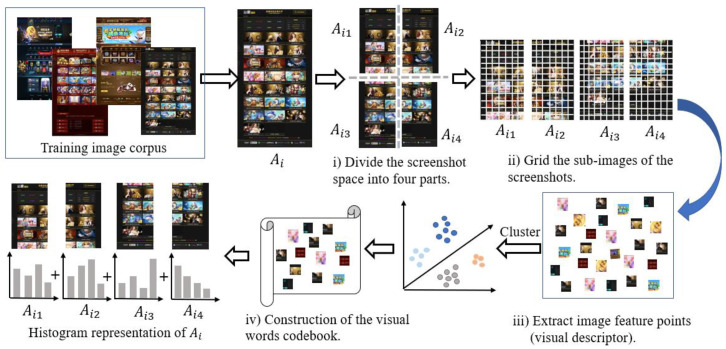
The process of the image representation using the Spa-BoVW.

To improve this situation, we integrated the local spatial relationship of the website screenshots into the BoVW model, proposing Spa-BoVW. The process of the image (website screenshots) representation using the Spa-BoVW is shown in Figure 3. The proposed Spa-BoVW divides the screenshot of the website into four sub-image blocks and considers the spatial relationship between the visual feature points among the four sub-blocks to capture more visual information. Furthermore, the inverse document frequency (IDF) was introduced to reduce the interference caused by visual stop words, which is beneficial for achieving more accurate and effective image feature representation. The specific implementations of image representation based on the Spa-BoVW model are as follows:

- Website screenshot segmentation

Here, we took the middle point of the four sides of the website screenshot and divided the screenshot into four sub-image blocks of equal size $A_{i}=A_{i1},A_{i2},A_{i3},A_{i4},$ where $A_{i}$ is the sample of the *i*th image screenshot and the spatial order of sub-images $A_{i1}$, $A_{i2}$, $A_{i3}$, $A_{i4}$ are shown in Figure 3. As already mentioned, for analyzing the complete visual information of the website, the long screenshot of the website was obtained by the website interface automatically scrolling down. Therefore, different websites may get different sizes of sub-images here.

- Keypoints extracting and keypoints description

The algorithm initially grids every sub-image of the screenshot, that is, all the sub-images are segmented into several small image blocks according to the preset grid size. Due to the different sizes of different website screenshots, the number of image blocks obtained by griding their corresponding subgraphs may be different. Then, feature extraction was performed on these image blocks. Speeded-up robust features (SURF) [50] and scale-invariant feature transform (SIFT) [51] are common feature extraction algorithms. However, compared with the SIFT algorithm, the SURF algorithm has better robustness for object rotation and illumination with a faster calculation speed [50]. Therefore, in this study, the SURF algorithm was used to extract the descriptors of the four sub-image samples of website screenshots. Each feature point (descriptor) was represented as a 64-dimensional feature vector and the image was represented as a set of image descriptors.

- Visual vocabulary construction

A visual vocabulary is constructed by clustering all feature descriptors extracted from the image training set. In the clustering process, all similar visual descriptors extracted in the training set are grouped. Due to the large number of SURF descriptors that are extracted from the image sample set, directly clustering these descriptors easily causes problems, such as memory overflow and long calculation times. For this reason, the Mini Batch K-Means [52] clustering algorithm was used to cluster all the descriptors. Different from K-means, the Mini Batch K-Means randomly extracts small-batch subsamples from the data set in each iteration instead of using the entire sample set, and updates the clusters using the method of convex combination, repeating this process until convergence. Therefore, this Mini Batch K-Means algorithm can improve the convergence speed of sample clustering and effectively alleviate the time-consuming effect caused by large sample sets; its effectiveness is demonstrated in References [53,54].

The Mini Batch K-Means clustering algorithm was used to cluster all the feature descriptors into *k* classes. After clustering, these *k* cluster centers were used as visual words and all visual words were used to construct the visual vocabulary codebook. The visual vocabulary codebook can be represented as $V=p_{1},p_{2},\ldots ,p_{k}$, where $p_{i}$ is the visual vocabulary and *k* is the number of visual words in the visual vocabulary codebook.

- Histogram representation of an image

First, for all SURF feature descriptors in the screenshot sub-image, we calculated the Euclidean distance between every feature descriptor and the words in the visual dictionary and map the feature descriptor to the closest visual word. The mapping rule can be represented as:

(1) $$\underset{\;p\in V}{Map\left(d_{i}\right)=argmin}\left(dist\left(p,d_{i}\right)\right),$$

where $dist\left(p,d_{i}\right)$ calculates the Euclidean distance between feature descriptor $d_{i}$ and visual word *p*, $Map\left(d_{i}\right)$ maps feature descriptor $d_{i}$ to visual word *p*, and V is the visual vocabulary codebook.

Then, the histogram was constructed to represent every screenshot sub-image by counting the word frequency of the visual vocabulary in the sub-image. In this way, the histogram could reflect the distribution of feature points extracted from the image in the visual dictionary. To reduce the interference caused by visual stop words, we calculated the IDF of the visual words and used it to weight the word frequency. Then the L2 normalization was performed on the obtained vector. The K-dimensional vector obtained after the above operations could be used to represent the sub-image. Finally, based on the order of the sub-image blocks $A_{i1}$, $A_{i2}$, $A_{i3}$, $A_{i4}$, the visual feature vectors of the sub-image were horizontally spliced together to obtain the visual feature representation of the final website screenshot.

### 3.4. Classification Based on the Image and Textual Features

**Figure 4:**
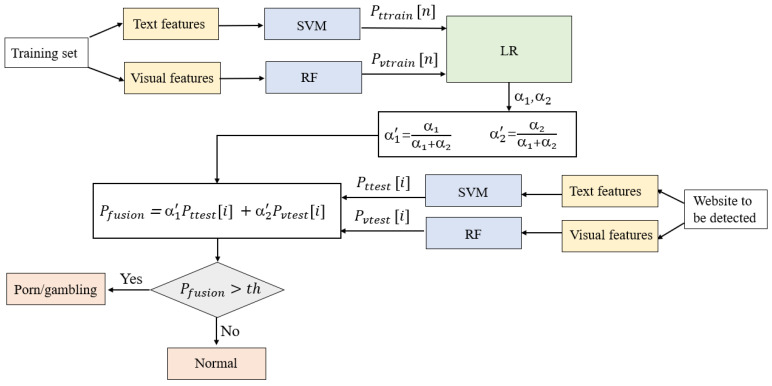
The flowchart of the classification algorithm.

In the classification stage, we proposed a classification method by combining SVM, RF, and LR, which enabled different models to be complementary. In this classification method, we designed a special category decision mechanism that comprehensively measured the influence of the textual features extracted from the website source code and image features extracted from the website screenshot on the reliability of the prediction. More specifically, this method mainly included two parts: classifier design and category decision. Figure 4 shows the flowchart of the final classification method, and the detailed steps of this method are described below.

First, we chose two classifiers (SVM and RF) to classify the extracted features in the classifier design part. In the textual-features-based classification, we used the textual feature vectors in the website training set obtained in Section 3.3.1 to train the SVM, which was used for making classification predictions for textual features of subsequent test sets. For the visual-features-based classification, the image feature vectors in the training set obtained in Section 3.3.2 were used to train the RF. After the above steps, we obtained four probability sets $P_{ttrain}\left[n\right]$, $P_{ttest}\left[m\right]$, $P_{vtrain}\left[n\right]$, and $P_{vtest}\left[m\right]$, where *n* and *m* are the numbers of websites in the training set and test set, respectively. More detailed definitions of the probabilities are as follows:

- $P_{ttrain}\left[i\right]\left(i\leq n\right)$ is an element in the probability set of $P_{ttrain}\left[n\right]$. $P_{ttrain}\left[i\right]$ is the probability that the *i*th website in the training set is predicted to be a pornographic or gambling website based on the text classification model (SVM).
- $P_{ttest}\left[i\right]\left(i\leq m\right)$ is an element in the probability set of $P_{ttest}\left[m\right]$. $P_{ttest}\left[i\right]$ is the probability that the *i*th website in the test set is predicted to be a pornographic or gambling website based on the text classification model (SVM).
- $P_{vtrain}\left[i\right]\left(i\leq n\right)$ is an element in the probability set of $P_{vtrain}\left[n\right]$. $P_{vtrain}\left[i\right]$ is the probability that the *i*th website in the training set is predicted to be a pornographic or gambling website based on the visual classification model (RF).
- $P_{vtest}\left[i\right]\left(i\leq m\right)$ is an element in the probability set of $P_{vtest}\left[m\right]$. $P_{vtest}\left[i\right]$ is the probability that the *i*th website in the test set is predicted to be a pornographic or gambling website based on the visual classification model (RF).

In the category decision module, a fusion algorithm based on LR was designed to fuse the results of two classifiers. The probability lists $P_{ttrain}\left[n\right]$ and $P_{vtrain}\left[n\right]$ that were obtained based on the training set were used as new two-dimensional feature vectors for training the LR to obtain the importance degree of the two features, which expresses the degree of influence on the classification results.

We used $\alpha_{1}$ and $\alpha_{2}$ to indicate the importance of features $P_{ttest}\left[i\right]$ and $P_{vtest}\left[i\right]$, respectively. To facilitate the subsequent weighting calculation, we mapped the values of $\alpha_{1}$ and $\alpha_{2}$ to the interval [0,1] and represent them as $\alpha_{1}^{\prime }$ and $\alpha_{2}^{\prime }$, which were calculated as follows:

(2) $$\alpha_{1}^{\prime }=\frac{\alpha_{1}}{\alpha_{1}+\alpha_{2}},$$

(3) $$\alpha_{2}^{\prime }=\frac{\alpha_{2}}{\alpha_{1}+\alpha_{2}},$$

where the $\alpha_{1}^{\prime }$ and $\alpha_{2}^{\prime }$ were also called weighting coefficients, with $\alpha_{1}^{\prime }+\alpha_{2}^{\prime }=1$.

For any of the websites in the test set, we defined a probability value $P_{fusion}$ that represented the probability of predicting the website to be a pornographic or gambling website after a classification decision considering both the textual and image features. The formula to compute $P_{fusion}$ was as follows:

(4) $$P_{fusion}=\alpha_{1}^{\prime }P_{ttest}{\left[i\right]}_{\;}+\alpha_{2}^{\prime }P_{vtest}\left[i\right].$$

It is obvious that the greater the weighting coefficient, the more significant the impact of the corresponding classification result on the $P_{fusion}$.

Finally, we designed a decision condition:

(5) $$P_{fusion}>th,$$

where th is a threshold we set artificially. If the condition is true, there was enough confidence to predict the website as a pornographic or gambling website. If not, then it was marked as a normal website.

After obtaining the final prediction category of the website, the system will add it to the URL blacklist or whitelist. By constantly updating the URL blacklist and whitelist, the system can make a quick judgment when detecting the same website in the future, thus improving the detection speed.

## 4. Experimental Results

In this section, we describe the design and process of the experiments to verify the effectiveness of the proposed method. First, we describe the dataset collected for our experiment. Then the experimental indicators used for evaluating the experimental results are described. Finally, the experimental design and result analysis are described in three parts to verify the effectiveness of our proposed method.

### 4.1. Experimental Dataset

To evaluate the proposed system’s performance, multiple sets of comparative experiments were conducted on a real-life dataset collected from the Internet. We collected data traffic between the user and the Web server from the mirror of the egress router and analyzed it using protocol analysis tools. The final website dataset consisted of a total of 2722 websites distributed among three categories, namely gambling, pornographic, and normal (including science, news, shopping, education, movie, entertainment, sports, etc.). Table 1 shows our experimental dataset, including 876 gambling websites, 862 porn websites, and 984 normal websites. Among them, 60% of the website dataset was taken as the training set and 40% was taken as the testing set.

### 4.2. Performance Metrics

In the following experiments, accuracy, precision, recall, and F-measure were used as performance metrics to evaluate the proposed approach. Table 2 shows the confusion matrix used in this part. The matrix was composed of four indicators: true positive (TP), true negative (TN), false positive (FP), and false negative (FN). Among them, TP is the number of gambling (pornographic) websites correctly predicted as gambling or pornographic websites, TN is the number of normal websites correctly predicted as normal websites, FP is the number of normal websites incorrectly predicted as gambling or pornographic websites, and FN is the number of gambling or pornographic websites incorrectly predicted as the normal websites. 

The performance metrics mentioned above are defined as follows. Accuracy measured the rate of illegal (gambling or porn) and normal websites that were predicted correctly as a proportion of all the websites:

(6) $$\mathrm{Accuracy}=\frac{\mathrm{TP}+\mathrm{TN}}{\mathrm{TP}+\mathrm{FP}+\mathrm{FN}+\mathrm{TN}}.$$

Precision measured the rate of illegal (gambling or porn) websites that were predicted correctly as a proportion of all websites predicted as illegal (gambling or porn):

(7) $$\mathrm{Precision}=\frac{\mathrm{TP}}{\mathrm{TP}+\mathrm{FP}}.$$

Recall measured the rate of instances correctly predicted as illegal (gambling or porn) as a proportion of all illegal websites (gambling or porn):

(8) $$\mathrm{Recall}=\frac{\mathrm{TP}}{\mathrm{TP}+\mathrm{FN}}.$$

The F-measure is the harmonic mean of the recall and the precision:

(9) $$F-\mathrm{measure}=2\times \frac{\mathrm{Precision}\times \mathrm{Recall}\;}{\mathrm{Precision}+\;\mathrm{Recall}\;}.$$

### 4.3. Experimental Design and Result Analysis

#### 4.3.1. Classification Based on Textual Features

In this part, we evaluate the performance of the website classification based on textual features using Doc2Vec and an SVM. Our first set of experiments explored the optimal performance settings of the Doc2Vec model. For this purpose, different Doc2Vec models were trained by combining different textual feature dimensions, model frames, and context window size parameter settings. First, we explored how the framework and text window sizes of the Doc2Vec could influence the classification efficiency. In the experiment, the text vector dimension was set to 200, while the text window was chosen from {5,10} and the framework was chosen from {DM, DBOW}. Table 3 provides a performance comparison of Doc2Vec using different text window and framework combinations on our pornographic and gambling website datasets.

From Table 3, we found that the Doc2Vec model trained with DBOW had a better performance in terms of all evaluation metrics than DM for both gambling and pornographic websites. The most notable was the recall, which increased by 4.85% for pornographic websites (window = 5). Furthermore, it was also shown that the smaller the window size, the better the performance of the Doc2Vec model, even though the recall dropped slightly (keeping the same framework). The reason might have been that textual information on a website was not as short and clever as blog posts, and therefore choosing a larger text window size means selecting more context words. This made it easy to mix in unnecessary redundant information, leading to a reduction in the classification effect. It was also noted that in all our datasets (gambling and porn), Doc2Vec with parameter settings of DBOW being selected and a context window size of 5 performed the best in terms of all the evaluation indicators; therefore, this parameter setting was used for subsequent experiments.

Next, we evaluated the impact of the dimensionality of the textual feature and built the best Doc2Vec model. In this experiment, the vector dimensions of 100, 200, 300, and 400 were considered. The performance comparisons of Doc2Vec using different vector dimensions on pornographic websites and gambling websites are shown in Table 4 and Table 5. As shown in Table 4, for the pornographic websites, when the vector size parameter was raised from 100 to 200, the performance of the Doc2Vec improved and then degraded when it was further raised to 400. The model with 200 dimensions achieved the best performance, with an accuracy of 91.94%, a recall of 89.14%, and an F1-measure of 91.23%. As shown in Table 5, similar to the pornographic websites, the performance of the classification on gambling website datasets showed a trend of first increasing and then decreasing, and reached the peak when the dimension was 300, with an accuracy of 90.39% and an F-measure of 89.39%. As a consequence, dimension = 200 was chosen as the optimal vector size parameter for the pornographic website classification, while dimension = 300 was chosen as the optimal vector size parameter for the gambling website classification.

Furthermore, to further illustrate the effectiveness of learning textual features using Doc2Vec, we also present the experimental results comparing the classification performance of feature representation using Doc2Vec and Word2Vec in Table 4 and Table 5. From the above experimental results, it can be observed that Doc2Vec yielded higher performance compared to Word2Vec in terms of all evaluation metrics. The most important reason was that Word2Vec only represents features based on word dimensions but does not consider the semantic relationship of the context. Doc2Vec introduces the semantic relationships of text based on Word2Vec; therefore, it was more suitable for our document-level text data set.

#### 4.3.2. Classification Based on Visual Features

The purpose of the experiments in this subsection was twofold. First of all, we varied the clustering center number to obtain the most effective Spa-BoVW model for image feature representation. The second and more important goal was to verify the effectiveness of Spa-BoVW by comparing its performance with that of traditional BoVW.

The cluster number is the number of visual words in the visual codebook and the dimension of the feature vector represented by the histogram of each screenshot sub-image in our experiment, which was the important influencing factor of the detection performance. In this set of experiments, we compared the performance of Spa-BoVW and BoVW using different cluster numbers on pornographic and gambling websites datasets, where the experimental results are shown in Table 6 and Table 7. The cluster numbers [100, 200, 300, 400, 500] were considered.

We first discuss the relationship between the number of clusters used in Spa-BoVW and the classification results. From the results in Table 6, it can be found that all the evaluation indicators increased first and then decreased when the cluster number increased. The best classification performance of pornographic websites was obtained when the number of clusters was 300, with the accuracy, precision, recall, and F-measure reaching 95.03%, 96.72%, 92.57%, and 94.60%, respectively. The results in Table 7 show that the classification performance of the gambling websites also peaked at cluster number = 300, where the accuracy, precision, and F-measure all exceeded 93%. By comprehensively analyzing the results of Table 6 and Table 7, it can be concluded that having a cluster number (visual vocabulary numbers) that was too high or too low was not conducive to the representation of visual features. This was because having fewer clusters resulted in feature points with a low similarity being assigned to the same cluster, while having too many clusters led to similar feature points being divided into different clusters. These all affected the representation of the cluster center, resulting in the reduction of the Spa-BoVW performance.

The experimental results reported in Table 6 and Table 7 also suggest that when keeping the same number of cluster centers, the proposed Spa-BoVW outperformed the traditional BoVW on each dataset with a large margin. For instance, for pornographic websites (see Table 6), the accuracy increased from 87.77% to 95.03% (an increase of 7.26%), while the precision increased from 88.43% to 96.72% (an increase of 8.29%), which was the most obvious improvement (cluster number = 200). As can be seen from Table 7, even in the case of the weakest improvement in the classification performance of gambling websites (cluster number = 500), all four evaluation metrics increased by at least 4.47%. As specified in Section 3.3.2, notwithstanding that traditional BoVW can extract visual feature points from screenshots for image classification, it has a well-known shortcoming in that it overlooks spatial information among extracted feature points, which ultimately affected the classification performance in this study. The excellent performance of Spa-BoVW can be mainly explained by the fact that the Spa-BoVW considered the positional relationship of feature points between space blocks, which was useful for capturing discriminative visual patterns and obtaining effective visual feature representations.

#### 4.3.3. Textual and Visual Combination-Based Classification

From the above experimental results presented in Section 4.3.1 and Section 4.3.2, it can be clearly seen that models using a single type of feature (textual or visual) cannot have a satisfactory classification performance. In this section, we try to comprehensively consider the textual features and visual features of the website in classification prediction to achieve an improvement in the detection performance of the website.

In the following experiments, we used the Doc2Vec and the Spa-BoVW with the optimal parameter settings. For pornographic websites, we chose Doc2Vec with model = DBOW, window = 5, and dimensions = 200 to extract the textual features and classify them using an SVM. Spa-BoVW with cluster number = 300 was used to extract the visual features and an RF was used for the visual features classification. For gambling websites, except for the dimension of the vector size of the Doc2Vec model being set to 300, the rest of the parameters of the Doc2Vec and Spa-BoVW models were consistent with those used for pornographic websites. Furthermore, the importance coefficient of the features (the probability of $p_{ttest}\left[i\right]$ and $p_{vtest}\left[i\right]$ in LR were used to visualize the weight coefficients $\alpha_{1}^{\prime }$ and $\alpha_{2}^{\prime }$, and the results for different types of websites obtained in our experiment are shown in Table 8.

From Table 8, we can see that for both gambling and pornographic websites, the weighting coefficient $\alpha_{2}^{\prime }$ was greater than the weighting coefficient $\alpha_{1}^{\prime }$. This means that the prediction result of the visual-features-based classification had a greater impact on the final classification. It can be seen that the weight coefficient $\alpha_{2}^{\prime }$ of pornographic websites was larger than that of gambling websites, which indicates that for pornographic website detection, the prediction probability of visual-features-based classification contributed more to the final prediction result. This may have been due to pornographic websites being more colorful and having a greater proportion of images compared to gambling websites. This made it possible to use Spa-BoVW extract more contrasting feature points from the screenshot of the pornographic website and thus was more conducive to distinguishing pornographic websites from normal websites.

Section 3.4 details our website classification decision method. In this method, the threshold parameter *th* played a crucial role in our decision process. The following experiments focused on exploring the influence of the threshold on the prediction performance of pornographic and gambling websites. The experiments were conducted by varying the threshold, whose values ranged from 0.2 to 0.6. Table 9 shows a comparison of the experimental results using different thresholds. It can be seen from Table 9, when the *th* = 0.4, the method obtained the best classification performance for pornographic websites, with the accuracy, precision, and F-measure all exceeding 99%. On the other hand, the classification method for gambling websites performed the best when *th* = 0.5, with the highest accuracy of 99.18% and the highest F-measure of 99.13%. It is worth mentioning that as the threshold increased, the accuracy continuously improved and the recall rate decreased. This was mainly because raising the threshold was equivalent to raising the requirement to predict websites as gambling or pornography websites, which reduced the likelihood of predicting a normal website as a gambling or pornography website, and therefore the accuracy went up. At the same time, there were more gambling or pornographic websites with nonobvious characteristics that were predicted to be normal websites, which reduced the recall rate.

**Figure 5:**
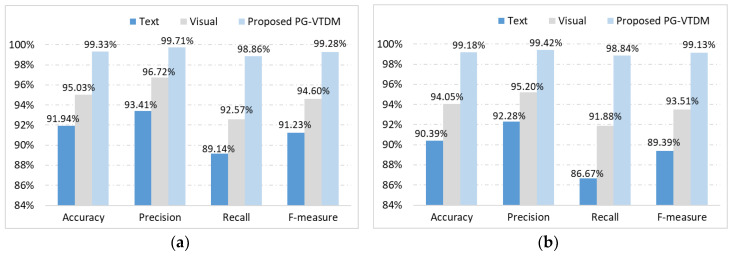
Performance comparisons between the single-feature-detection methods and the proposed PG-VTDM: (**a**) pornographic websites dataset and (**b**) gambling websites dataset.

To verify the effectiveness of the proposed PG-VTDM, a performance comparison between the best single-feature-detection methods (discussed in Section 4.3.1 and Section 4.3.2) and PG-VTDM for each dataset is shown in Figure 5. It can be seen from Figure 5 that the classifier combination algorithm based on a category decision mechanism performed significantly better at detecting gambling and pornography websites. Compared with the best visual-features-based models, the combined-feature model achieved better recall values by 6.29% (porn) and 6.96% (gambling), and better F-measure values by 4.68% (porn) and 5.62% (gambling). Similarly, the combined-feature model achieved better recall values by 9.72% (porn) and 12.17% (gambling), and better F-measure values by 8.05% (porn) and 9.74% (gambling) compared with the best textual-features-based model. In summary, our method with the category decision mechanism that takes advantage of the complementarity between textual and image features significantly improved the classification performance of detecting illegal websites.

Table 10 shows the detection time of the proposed system for different types of websites, which includes the time spent in the process of blacklist and whitelist filtering, website content acquisition, feature extraction, and the final classification prediction. As can be seen from Table 10, no matter what type of website detection, the longest detection time of the system does not exceed 1.157s and the shortest time was as low as 0.427s, with the average detection time being within 1 s. Based on the above results, the proposed system is believed to be capable of real-time detection.

Furthermore, the performance of the proposed method was compared with some state-of-the-art porn and gambling website detection approaches. We simulated the method used in References [9,21] on our experimental set and compared their performance with that of our proposed method in Table 11. The approaches from References [9,21] analyze website features from the perspective of textual content [21] or visual content [9]. From Table 11, it can be observed that our proposed approach outperformed other approaches for all testing indicators. Compared with these approaches, our method showed superior performance, mainly because the detection process relied on features from multiple aspects, which enabled a more comprehensive analysis of gambling and pornography websites, thus improving the recognition rate of these websites. The design of the decision mechanism also made the prediction result more reliable.

In addition, we also analyzed some possible reasons why the performance metrics did not reach 100%. For instance, some textual content on the website data set was relatively short and informal, thus the effect was slightly reduced when using Doc2Vec for textual content analysis. Moreover, a relatively small amount of training data may have affected the performance of Doc2Vec to a certain extent, and we expect to alleviate this problem by collecting more data sets in the future. There were also some unavoidable reasons caused by human operations, such as insufficient model tuning and errors in manual labeling.

## 5. Conclusions and Future Work

In this study, an automatic detection system for porn and gambling websites based on visual and textual content using a decision mechanism (PG-VTDM) was proposed. Deploying this system on the intelligent wireless router at home or school can effectively protect children and teenagers from the adverse effects of exposure to gambling and pornography websites, which could be applied in other domains for specific purposes via obtaining HTML source codes and website screenshots. We used two sorts of features, namely, the textual features extracted from the HTML source code and the visual features extracted from the website screenshots, which were independent of third-party services, enabling the proposed method to detect in real-time. In particular, Spa-BoVW, which was obtained by introducing spatial relations into the traditional BoVW, was designed to achieve a more complete and representative visual feature representation of website screenshots. The proposed decision mechanism combined the classification results of a text-based classifier and image-based classifier using a fusion algorithm and enabled the final prediction results to be more accurate and reliable than its competitors. By analyzing the textual and visual features of the websites, this method can bypass the methods illegal website builders use to evade the detection of websites though disguising the text or pictures of websites as normal information, which can greatly improve the detection speed, while also ensuring the detection performance via maintaining the blacklist and whitelist libraries. The experimental results indicate that compared with some advanced systems, the proposed method was very efficient at the detection of porn and gambling websites, with all the performance metrics reaching satisfactory standards.

Furthermore, the proposed detection framework only needed to analyze the textual and visual content of the websites that are common and easily obtained for all types of websites. Therefore, using this detection framework in other detecting domains is feasible and effortless. The proposed framework provides comprehensive, complete, and detailed modules with a strong universality, which leads to the extensibility and portability of this proposed framework. In future work, we plan to collect more types of website samples, such as fraud websites, terrorist websites, and drug trade websites, extending this system to more types of website recognition scenarios. In addition, deploying our system on resource-constrained devices and exploring more valuable features to achieve more accurate detection will be taken into consideration in the future.

## Figures and Tables

**Table 1:** Experimental dataset.

Website Classes	Total Websites	Training Sets	Testing Sets
Porn	876	526	350
Gambling	862	517	345
Normal	984	590	394

**Table 2:** The confusion matrix

	Actual Value	Actual Gambling (Porn)	Actual Normal Websites
Predict Value			
**Predicted as gambling or porn**		TP	FP
**Predicted as normal**		FN	TN

**Table 3:** Performance of Doc2Vec using different text window and framework combinations.

Dimensions	Framework	Window	Accuracy (%)	Precision (%)	Recall (%)	F-Measure (%)
			Porn|Gambling	Porn|Gambling	Porn|Gambling	Porn|Gambling
200	DM	5	89.38|87.82	92.48|90.22	84.29|82.89	88.20|86.40
200	DM	10	88.58|86.74	89.79|87.54	85.43|83.48	87.56|85.46
200	DBOW	5	91.94|89.58	93.41|91.88	89.14|85.22	91.23|88.42
200	DBOW	10	90.99|88.23	91.25|88.17	89.42|86.38	90.33|87.27

**Table 4:** Performance of Doc2Vec vs. Word2Vec for pornographic website classification.

Dimensions	Accuracy (%)	Precision (%)	Recall (%)	F-Measure (%)
	Doc2Vec|Word2Vec	Doc2Vec|Word2Vec	Doc2Vec|Word2Vec	Doc2Vec|Word2Vec
100	91.26|7.50	92.79|88.13	88.29|84.86	90.48|86.46
200	91.94|87.90	93.41|88.92	89.14|85.14	91.23|86.99
300	91.53|88.31	94.15|89.25	87.42|85.43	90.66|87.30
400	90.86|86.96	92.99|87.99	87.14|83.71	89.97|85.80

**Table 5:** Performance of Doc2Vec vs. Word2Vec on gambling website classification.

Dimensions	Accuracy (%)	Precision (%)	Recall (%)	F-Measure (%)
	Doc2Vec|Word2Vec	Doc2Vec|Word2Vec	Doc2Vec|Word2Vec	Doc2Vec|Word2Vec
100	89.17|87.01	91.54|88.79	84.64|82.61	87.95|85.59
200	89.58|88.90	91.87|90.22	85.22|84.35	88.42|87.65
300	90.39|88.63	92.28|91.17	86.67|83.77	89.39|87.31
400	89.85|87.69	92.72|89.94	84.93|82.90	88.65|86.28

**Table 6:** Performance of Spa-BoVW vs. BoVW at detecting pornographic websites.

Cluster Number	Accuracy (%)	Precision (%)	Recall (%)	F-Measure (%)
	Spa-BoVW|BoVW	Spa-BoVW|BoVW	Spa-BoVW|BoVW	Spa-BoVW|BoVW
100	92.61|86.83	93.77|87.28	90.29|84.29	92.00|85.59
200	94.09|87.50	95.81|87.91	91.43|85.14	93.57|86.50
300	95.03|87.77	96.72|88.43	92.57|86.96	94.60|87.69
400	93.81|88.31	95.50|89.02	91.14|85.71	93.27|87.34
500	92.47|87.63	93.49|88.62	90.29|84.57	91.76|86.55

**Table 7:** Performance of Spa-BoVW vs. BoVW in detecting gambling websites.

Cluster Number	Accuracy (%)	Precision (%)	Recall (%)	F-Measure (%)
	Spa-BoVW|BoVW	Spa-BoVW|BoVW	Spa-BoVW|BoVW	Spa-BoVW|BoVW
100	90.93|85.12	91.87|85.71	88.41|81.74	91.11|83.43
200	92.29|85.52	93.11|86.06	90.15|82.32	91.61|84.49
300	94.05|86.06	95.20|86.45	91.88|83.19	93.51|84.79
400	93.10|86.87	94.54|87.13	90.43|84.35	92.44|85.72
500	91.75|87.28	92.77|87.69	89.28|84.64	90.99|86.14

**Table 8:** Weighting coefficients $\alpha_{1}^{\prime }$ and $\alpha_{2}^{\prime }$ for website detection.

Website	Weighting Coefficient $\alpha_{1}^{\prime }$	Weighting Coefficient $\alpha_{2}^{\prime }$
Pornographic	0.3	0.7
Gambling	0.4	0.6

**Table 9:** Experimental results using different thresholds (*th*).

Threshold (*th*)	Accuracy (%)	Precision (%)	Recall(%)	F-Measure (%)
	Porn|Gambling	Porn|Gambling	Porn|Gambling	Porn|Gambling
0.2	96.64|94.45	93.33|89.38	100|100	96.55|94.39
0.3	98.52|97.29	97.21|94.52	99.71|100	98.30|97.18
0.4	99.33|98.65	99.71|97.72	98.86|99.42	99.28|98.56
0.5	97.98|99.18	100|99.42	95.71|98.84	98.11|99.13
0.6	94.62|97.97	100|100	88.57|95.65	93.94|97.78

**Table 10:** Detection time of the proposed system.

Website	The Shortest Time (s)	The Longest Time (s)	The Average Time(s)
Pornographic	0.552	1.157	0.843
Gambling	0.427	0.978	0.705

**Table 11:** Performance comparisons between the proposed PG-VTDM and the existing state-of-the-art approaches.

Method	Accuracy (%)	Precision (%)	Recall (%)	F-Measure (%)
	Porn|Gambling	Porn|Gambling	Porn|Gambling	Porn|Gambling
Li et al. [9]	93.41|92.15	93.62|92.33	92.29|90.72	93.51|92.24
Sheu et al. [21]	97.45|96.07	97.69|96.20	96.86|95.36	97.57|96.13
Our proposed PG-VTDM	99.33|99.18	99.71|99.42	98.86|98.84	99.28|99.13
